# Post-COVID-19 Cognitive Dysfunction: Analyzing the Role of Age, Lifestyle, and Neurological Impairments: A Multi-Centric Case-Control Study

**DOI:** 10.2174/0117450179395261251006055231

**Published:** 2025-10-31

**Authors:** Samar A. Amer, Ines F. Djelleb, Ehab M. Ishteiwy, Mostafa Meshref, Youmna A. Amer, Jaffer Shah, Mahmoud Tarek Hefnawy, Noha A. Hashim, Carlos Schönfeldt-Lecuona, Mohamed E.G. Elsayed, Eman F. Ali

**Affiliations:** 1 Department of Public Health and Community Medicine, Faculty of Medicine, Zagazig University, Zagazig, Egypt; 2 Faculty of Medicine, Badji Mokhtar University, Annaba 23000, Algeria; 3 Albayda Medical Centre (AMC), Omar-Almukhtar University, Albayad, Libya; 4 Neurology Department, Faculty of Medicine, Alazhar University, Cairo, Egypt; 5 Department of Rheumatology and Rehabilitation, Faculty of Medicine, Zagazig University, Zagazig, Egypt; 6 Medical Research Centre, Kateb University, Kabul, Afghanistan; 7 Faculty of Medicine, Zagazig University, Zagazig, Egypt; 8 Neurology Department, Faculty of Medicine, Zagazig University, Zagazig, Egypt; 9 Department of Psychiatry and Psychotherapy III, University of Ulm, Leimgrubenweg 12-14, 89075 Ulm, Germany; 10 Department of Psychiatry, School of Medicine and Health Science, Carl von Ossietzky University Oldenburg, Oldenburg, Germany; 11 Department of Psychiatry, Faculty of Medicine, Zagazig University, Zagazig, Egypt

**Keywords:** Cognitive impairment, Post-COVID-19 syndrome, Physical activity, Dietary supplements, Olfactory dysfunction, Brain food diet, The Montreal Cognitive Assessment

## Abstract

**Introduction:**

The effects of COVID-19 extend beyond acute illness, with many survivors experiencing persistent symptoms. This study aimed to determine the frequency and contributing factors of cognitive impairment and other neurological symptoms in COVID-19 survivors four weeks after diagnosis, compared with healthy controls during the pandemic's fourth wave.

**Methods:**

A multicenter case-control study was conducted involving 176 COVID-19 survivors, diagnosed four weeks prior, and 92 healthy controls from Algeria, Egypt, and Libya. Data were collected through interviews using a structured, validated questionnaire administered by a trained physician.

**Results:**

Post-COVID-19 survivors exhibited significant cognitive deficits, chronic fatigue, and sensory impairments (including loss of appetite, taste, smell, and hearing). Cognitive impairment (Montreal Cognitive Assessment [MoCA] score <26) was observed in 57 participants (32.3%), with those affected being older (44.6 ± 16.9 years, *P* < 0.001) and consuming more junk food (8.6 ± 3.3 servings, *P* = 0.04). Cognitive disorders were more frequent among females (83.3%), smokers (57.9%), highly educated individuals (76.5%), and married participants (63.7%).

**Discussion:**

The study revealed a substantial burden of cognitive and sensory impairments in post-COVID-19 patients, supporting global observations and emphasizing the need for early screening and lifestyle interventions. The reliance on self-reported data and a case-control design limit causal inference.

**Conclusion:**

Post-COVID-19 survivors showed significant cognitive deficits, fatigue, and sensory impairments. Cognitive impairment was present in 32.3%, with higher prevalence in females, smokers, highly educated individuals, and married participants.

## 1 INTRODUCTION

As of May 11, 2022, the World Health Organization (WHO) reported 518,480,076 confirmed cases of coronavirus disease 2019 (COVID-19) [1]. COVID-19 represents a threat that transcends acute disease management, encompassing medium- and long-term health implications. A significant proportion of adults and children with COVID-19, ranging from 40-90%, have reported ongoing symptoms associated with the infection [2].

Post-COVID-19 conditions encompass a range of physical, social, and psychological effects resulting from the prior infection. This condition varies, with symptoms that persist, new symptoms that appear later, or changes in symptoms that occur one month after infection, even in people who did not have any symptoms [3]. Post-COVID-19 syndrome is characterized as a newly emerging chronic disease that impacts various systems and may lead to a combination of health conditions with differing durations [4].

The core symptoms of post-COVID-19 syndrome encompass post-exertional malaise, arthralgia, myalgia, and disturbances in the senses of smell, appetite, hearing, taste, and emotional perception, as well as sleep disturbances [5-8]. Additionally, about 20% of recovered COVID-19 patients and 33% of current COVID-19 patients have experienced one or more cognitive impairments. These impairments could include memory problems, attention difficulties, or dysexecutive syndrome (shown by symptoms such as not paying attention, getting lost, or moving in a disorganized way when instructed) [9, 10].

Cognitive impairment following COVID-19 affects the quality of life for millions and contributes to a substantial global economic burden; however, unlike other post-COVID-19 symptoms, there are currently no specific and effective treatments available [11].

The precise pathophysiological mechanisms underlying post-COVID-19 psychiatric and neurological consequences remain unclear; however, several neurotoxic mechanisms have been proposed. Encephalitis occurs when a virus penetrates the central nervous system (CNS) either directly through the blood-brain barrier or indirectly through the axonal transmission of olfactory neurons [12]. This process damages and disables neurons. Coagulopathies and disorders of cerebral blood vessels contribute to the occurrence of hemorrhagic or ischemic strokes. Moreover, peripheral organ dysfunction and excessive systemic inflammatory responses adversely affect brain function [13-17].

Arab nations adopted early and significant precautionary measures to address the severe acute respiratory syndrome coronavirus-2 (SARS-CoV-2) pandemic and its enduring effects. The primary objective of this study was to determine the frequency of cognitive impairments, defined as a MoCA score of less than 26, following COVID-19 compared to a well-matched control group.

The secondary objectives were to explore the determinants of post-COVID-19 cognitive impairments, including brain-healthy diet, physical activity levels, and supplement intake, among individuals who recovered from COVID-19 four weeks post-diagnosis during the fourth wave of the pandemic, compared to a well-matched control group of apparently healthy individuals in Egypt, Algeria, and the Arab Republic of Libya.

## 2 METHODOLOGY

### 2.1 Research Design And Setting

Our study was designed as a multi-centric case-control study and included data acquired in three Arab countries (Egypt, Libya, and Algeria) during the fourth wave of the COVID-19 pandemic. Data acquisition took place from May 11, 2022, to June 11, 2022.

### 2.2 Sample Size And Sampling Techniques

The sample size was estimated using the Epi Info^TM^ version 7 program. Given the limited data on the prevalence of post-COVID-19 chronic fatigue syndrome (CFS) and cognitive impairments, we relied on a previous study [18]. This study reported that nearly 20% of infected COVID-19 patients had one type of cognitive impairment, with a 95% confidence level and 80% power. The calculated sample size was 90 (60 cases and 30 controls) from each country. We employed a stratified random sampling method for this purpose. The sample size was stratified according to age into 20-year intervals in each group, forming the following age groups: 18 to less than 35 years, 35 to less than 55 years, and 55 to less than 75 years from each country.

### 2.3 Participants

Selection criteria: Patients were 18 years or older, with confirmed Polymerase Chain Reaction (PCR) –positive SARS-CoV-2 infection for more than four weeks. We excluded patients who died before follow-up, were mentally unfit, had autoimmune diseases, had a previous history of complicated neurophysiological disorders, were unable to move freely due to concomitant osteoarthropathy, or were immobile before or after COVID-19 due to diseases such as stroke or pulmonary embolism.

The cases were well matched with controls based on education level, residence, occupation, marital status, and family history in each country. This study included 92 age- and sex-matched healthy individuals who served as controls. All patients and control groups underwent a full neurological assessment, with particular attention to the senses of smell, hearing, and taste.

### 2.4 Data Collection Tool

#### 2.4.1 The Development And Validation Of The Data Collection Tool

A well-designed Arabic-English questionnaire was used to gather the data in the supplementary material. The survey was developed and adopted using information from previous studies [18-21]. The English version of the questionnaire was translated into Arabic by a bilingual panel consisting of two healthcare professionals and one externally qualified medical translator independently completed. Two English-speaking translators independently completed the back-translation, consulting the original panel for any issues.

To ensure consistent answers across the three countries, we assessed the questionnaire's reliability and validity. Four doctors, two neurologists, one rheumatologist, and one psychiatrist, one from each country, checked that the questionnaire was correct. It was then read and understood by 15 people from each region in a pilot study whose results were not included. The questionnaire was found to be valid (Cronbach's alpha = 0.83).

#### 2.4.2 The Structure Of The Data Collection Tool

The data were collected through an interview and clinical examination by a well-trained physician after informed consent was completed (face-to-face interview). The following sections comprise the questionnaire:

### 1 Request For Respondent's Consent

### 2 Sociodemographic And Health-Related Variables

Age (in years), body mass index (BMI) [body weight (kg)/height in meters^2^], educational level, sex, nationality, occupation, marital status, family history of diseases and comorbidities.

### 3 The Lifestyle Determinants

###  I) Evaluation Of Nutritional Value (Brain-Healthy Diet And Dietary Supplement Consumption)

The brain-healthy diet includes healthy foods such as [omega-3 fatty acids (from nuts, fish, and tuna), vegetable oil, olive oil, caffeine (from one cup of coffee or more than two cups of tea), and strawberries or grapes], and unhealthy foods, including processed or ultra processed meat (hamburgers, hotdogs), fried foods, and sugar, are unhealthy brain foods. The total score for the brain food diet was determined using the Likert scale (never = 0, rarely = 1, occasionally = 2, frequently = 3, always = 4) [22-24]. Dietary supplement use was recorded (vitamin B12, folic Acid, iron, multivitamins, vitamins B/C/D/E, omega-3, zinc, and collagen).

### II) Level of Physical Activity

It was measured by the average number of steps taken over the past week (using pedometers or accelerometers *via* smartphones or watches or by self-report): The level of physical activity was classified as sedentary (less than 5,000 steps per day), low (5,000– less than 7,000 steps per day), average (7,000–10,000 steps per day), or high (more than 10,000 steps per day) [25].

### 4 The Information about COVID-19 Vaccinations and Past SARS-CoV-2 Infections

Including vaccine type and any adverse events (AEs)—was organized based on the latest updates from the Centers for Disease Control and Prevention (CDC) as of January 12, 2022 [4, 26]. The list includes no AEs; Local AEs (injection site) (such as pain, heaviness, redness, swelling); General AEs; and Systemic AEs. History of SARS-CoV-2 infection, treatment plan, symptom resolution, and their relationship to vaccination status were documented.

### 5 Neurological Assessment

Neurological assessment was conducted as follows:

### • Taste Impairment:

An objective taste test was conducted to determine gustatory function. Simple equipment for testing included tasting sprays of sucrose (1 g in 10 ml of water), citric acid (0.5 g in 10 ml of water), sodium chloride (0.75 g in 10 ml of water), and quinine hydrochloride at concentrations exceeding the thresholds for sweet, sour, salty, and bitter taste qualities. The patient was then directed to place two to three drops of the solution on their tongue. We then asked the participant, using a forced-choice paradigm, whether the spray was sweet, sour, salty, or bitter.

The participants were permitted up to three spray samples. If a participant failed to accurately identify two or more of the four sprays, they were considered to have gustatory dysfunction. In the general population, taste quality confusion is common, so failing to identify one spray was not considered pathological. The participants were required to identify the flavor in a different forced-choice paradigm. The tongue can only detect sweet, salty, sour, bitter, and “savory” (umami) tastes [27, 28].

### • The Chronic Fatigue Syndrome (CFS) Questionnaire

 was scored based on the subjects’ self-reported symptoms. Respondents rated their level of fatigue over the past six months using an anchored ordinal scale from 0 (no symptom) to 4 (severe). Eight additional criteria were used as substitutes for fatigue, including pain, headaches, myalgia, arthralgia, sore throat, lymph node swelling, cognitive dysfunction, and sleep disturbances. Based on the sum of the eight auxiliary criteria, each patient was classified as follows: Normal (fatigue = none, trivial, or mild; a score of 14), chronic idiopathic fatigue (CIF; fatigue = moderate or severe; a score of 14), CFS-like with insufficient fatigue syndrome (CFS-like; fatigue = none, trivial, or mild), and CFS [11, 29].

### • Olfactory (smell) Function Testing

I t was informed by an otolaryngologist using Sniffin' Sticks that were available on-site. Hummel et al.'s earlier report described Sniffin' Sticks as scented felt-tip pens [30]. The threshold and discrimination subtests randomly present individuals with three pens. In the identification test, the participant selected the appropriate olfactory descriptor from a list of four. Every 30 seconds, both nostrils receive Sniffin' Sticks, each lasting approximately 3 seconds.

Most locations used the Sniffin' Sticks test, which has three subtests and provides scores for odor threshold (1–16), discrimination (0–16), and identification. We combined the three scores to form a global olfactory function score, which includes threshold, discrimination, and identification (TDI score). All sites (n = 95) except one utilized the TDI test. The TDI score thresholds were applied for anosmia (16), hyposmia (30.5), and normosmia (>30.5). The cut-off values for this test were anosmia (6), hyposmia (7–10), and normosmia (score 11) [15, 31].

### • Cognitive Assessment Instrument

The Montreal Cognitive Assessment (MoCA) was used to assess multiple cognitive domains in the participant’s native language. It is a 30-question test. The test uses a scoring system that ranges from 0 to 30. A score of 26 or above is considered normal. The average score for individuals with mild cognitive impairment (MCI) **is** 22.1. The average score for Alzheimer's disease patients **is** 16.2 [20].

**Orientation** (6 Points, Maximum Score Achievable)

The test administrator requested participants to provide the date, month, year, day, location, and city.

**Short-term Memory/Delayed Recall** (5 Points, Maximum Score Achievable)

Five words were read; the participant was asked to repeat them. After completing further tasks, the participant was asked to repeat each of the five words. We informed them of the category to which the term belongs if they were unable to recall it.

**Executive Function/Visuospatial Ability** (5 Points, Maximum Score Achievable)

 The Trails B Test evaluates these two skills. The participants were required to draw a line connecting alternating numbers and letters in sequence and to sketch a cube.

*Language:* The participants were required to repeat two sentences correctly. Then we requested a list of all the terms in the sentences that begin with “F.” *Abstraction*: to evaluate abstract logic (2 points, maximum score to be achieved). We asked the participants to explain the similarities between two items, like a locomotive and a bicycle. *Animal naming* is a verbal fluency test worth 3 points, which is the maximum score that can be achieved. We showed three pictures of animals and then asked the person to name each one. *Attention (6 points,* maximum score to be achieved): The test-taker was asked to repeat a series of numbers forward and then a different series backward. *Clock-drawing test:* The person was asked to draw a clock that reads ten past eleven. *Education level*: 1 point was added to the test-taker's score if they had 12 years or fewer of formal education.

### 2.5 Statistical Analysis

The data were coded and analyzed using IBM SPSS Statistics, version 25 (IBM Corp., Armonk, NY, USA) and Stata, version 12 (StataCorp LLC, College Station, TX, USA), Stata Statistical Software, College Station, TX, Stata Corp., statistical significance was set at p < 0.05. After cleaning the data, descriptive statistics such as frequency and percentage were used to summarize the qualitative data, while quantitative data were presented as mean, SD, median, and range. A chi-squared test was used to assess the association between categorical variables. Moreover, the t-test, ANOVA (Analysis of Variance), and Kruskal-Wallis tests were used to assess the association between quantitative variables. In addition, Pearson's correlation coefficient (r) was used to evaluate the association between two continuous variables.

The cases were well-grouped and matched with controls based on age, sex, education level, residence, occupation, marital status, and family history in each country. Therefore, a simple logistic regression analysis within the framework of a generalized linear model was used to examine the association between other potential factors studied, with simultaneous adjustment in the multiple regression analysis.

To select a multivariable model with cognitive disorders (MoCA < 26) as the outcome variable, first analyze and understand the distribution of both the independent (participants’ hospitalization, comorbidities, and several symptoms at baseline) and outcome variables using univariate statistics. 2) Examine implausible values, significant interval variable deviations from the normal distribution, gaps, and outliers. 3) Compare the independent and outcome variables using bivariate analysis. 4). Determine if interval-independent factors affect outcomes linearly or convert, utilize splines, or generate dichotomous variables, then run a correlation matrix. We retained the significant factors (p < 0.05) (participants’ comorbidities, number of symptoms and signs at the onset of infection, admission to the hospital for SARS-CoV-2 infection, and interaction of the number of symptoms with hospital admission) in the model and iteratively tested all non-significant variables from the final model for possible significance in subsequent steps.

We fitted a final logistic regression model using a stepwise method to examine the independent associations of each potential factor with the outcome of interest. In this method, all factors that were significant (p < 0.05) in the univariable analysis were first added to the model. The data-model fit was then evaluated using the F test, likelihood ratio test, adjusted R^2^, Hosmer–Lemeshow test, c index, and the covariates’ outcome-estimating power. The likelihood ratio tests, along with adjusted odds ratios (aOR) and 95% confidence intervals (CIs), were used to examine the statistical significance of each factor.

### 2.6 Potential Bias

To minimize bias, we included PCR-confirmed COVID-19 cases, used validated tools (MoCA, Sniffin’ Sticks), and employed trained physicians for standardized assessments. Stratified random sampling and demographic matching helped reduce selection bias. A pilot-tested, back-translated questionnaire ensured reliability (Cronbach’s α = 0.83), and residual confounding was addressed through multivariate regression.

## 3 RESULTS

### 3.1 Sociodemographic, Health-Related, And Lifestyle Determinants Among The Studied Groups

Table **1** revealed no statistically significant differences between the case and control groups regarding demographics, BMI, or family history of the disease. However, Table **1** showed statistically significant differences between the case and control groups in terms of smoking, total brain food consumption score, and physical activity. More than half of the cases took dietary supplements, with 33.7% taking vitamins B, C, D, and E.

### 3.2 The History of SARS-CoV-2 Infection and the COVID-19 Vaccinations Among the Studied Groups

Table **2** showed that 56% of the cases had not been vaccinated, and 6.8% had received three doses of the vaccine. Among the COVID-19 cases, 82.4% reported remission of symptoms, while 1.1% reported persistent symptoms. Additionally, 86.4% of patients were treated at home. Since the SARS-CoV-2 infection, 2–26 months have elapsed.

There was a statistically significant difference between the case and control groups in terms of adverse events (AEs) after the COVID-19 vaccine. Of the cases, 31.6% experienced systemic manifestations after the vaccine, compared to 8.3% of the control group. However, there was no statistically significant difference between the two groups regarding COVID-19 vaccination status or the number of vaccine doses received.

### 3.3 The Neurological Assessment Among The Studied Groups

As shown in Table **3** and Fig. (**1**), there were statistically significant differences between case and control groups in terms of 18 (10.3%) cases of hearing impairment, 57 (32.3%) cases of cognitive impairment, 44 (25.0%) cases of taste impairment, 34 (19.3%) cases of appetite impairment, a lower overall cognitive score of 26.0 ± 3.76 with *p* = 0.001, and 74 (42.5%) cases of smell impairment.

SARS-CoV-2 infection was significantly associated (*p* = 0.001) with an increased risk of taste, appetite, smell, and hearing impairments in the case group compared to the control group, with approximate increases of 30, 4, 33, and 3 times for each impairment, respectively.

### 3.4 The Frequency And Determinants Of Neurological Impairments Among COVID-19 Cases

The frequency of cognitive impairment was 57 (32.3%) among cases. Compared to COVID-19 recovered patients without cognitive impairment, the frequency of post-COVID-19 cognitive impairment was significantly higher among females (83.3%), older age groups (36.8%), passive smokers or smokers (21.8%), high school/university students (76.5%), retirees (41.2%), and singles (34.3%).

According to the neurological assessment, post-COVID-19 patients exhibited statistically significant neurological deficits, indicated by a lower overall cognitive score of 26.0 ± 3.76 (*p* = 0.001), and they demonstrated a significantly higher fatigue scale score compared to the control group (*p* = 0.04). They also had significant impairments of appetite (*p* < 0.001), taste (*p* < 0.001), smell (*p* < 0.001), and hearing (*p* = 0.02) compared to the matched healthy controls (Tables **4** and **5**).

As shown in Table **6**, there was no statistically significant association between cognitive impairment among recovered COVID-19 cases and history of SARS-CoV-2 infection, current vaccination status, or associated neurological impairments. There was a moderately positive and significant correlation between the total Montreal Cognitive Assessment score and the presence of other neurological symptoms.

## 4 DISCUSSION

The clinical signs of SARS-CoV-2 infection across several organs cause post-acute sequelae of functional impairments in psychological, physical, and social domains of PCCs. PCCs, a condition that has recently emerged after COVID-19, have developed into long-term health complications, including cognitive impairment, with adverse impacts on individual health and well-being [32-35]. This study found no statistically significant differences in sociodemographic variables (age, sex, BMI, or family history of disease) between the case and control groups. Post-COVID-19 cases exhibited a higher prevalence of neurological disorders, including hearing, smell, taste, appetite, cognition, and chronic fatigue syndrome, compared to healthy controls. All these findings will be discussed in detail later.

### 4.1 Sociodemographic, Health-Related, And **Lifestyle Determinants Among The Studied Groups**

The case and control groups exhibited a statistically significant difference in the incidence of systemic adverse events (AEs) after the COVID-19 vaccine. Specifically, 31.6% of the patients experienced systemic AEs, compared to 8.3% of the control group. This finding is consistent with other studies showing that participants previously infected with SARS-CoV-2 had a higher incidence of systemic AEs after receiving the first doses of Pfizer (1.6-fold) and AstraZeneca (2.9-fold) vaccines compared to those without a known history of infection [36-39].

Regarding the self-reported resolution of COVID-19 symptoms, the majority of cases (145; 82.4%) reported resolution, while only two (1.1%) reported persistent symptoms. Over 40% of individuals previously infected with COVID-19 exhibited neurological impairments on clinical examination [4]. This is attributed to the pattern of post-COVID conditions (PCs), which can include persistent symptoms, new-onset late sequelae, or the evolution of symptoms experienced one month after infection, even among those who were initially asymptomatic. Most individuals were unaware of the connection between their neurological impairments and the SARS-CoV-2 infection.

This probability was lower than the rate observed in Saudi Arabia (SA), where 79.4% of participants continued to experience symptoms four weeks after disease onset [40]. In the present study, the median duration was nine months. Exhaustion or physical weakness (63%), anxiety or depression (23%), and difficulty resting (26%) were the most frequently reported symptoms, all associated with ME/CFS [41].

Most acute COVID-19 cases (152; 86.4%) were treated at home, while 15 (8.5%) were hospitalized. Only 9 (5.1%) required intensive care unit (ICU) care. This finding is comparable to research conducted in Germany between May 1 and November 30, 2020, in which only 6% of patients required hospitalization and 4% required ICU admission [42]. In Saudi Arabia (SA, January 2021), there were 68 (14.2%) hospital admissions.

Regarding the Lifestyle Determinants, this study exhibited statistically significant differences in brain food scores, smoking, and physical activity among the studied groups. For instance, the control group had a significantly lower frequency of smoking (2.2%), compared to 9.1% among COVID-19 cases. However, a unique German investigation found that smokers accounted for only 2% of the cases [43].

The dietary consumption of vitamins B, C, D, and E was significantly higher *(p* = 0.01) among COVID-19 cases; more than half of these cases took dietary supplements, with 33.7% specifically taking vitamins B, C, D, and E, compared to the control group. In accordance with other studies, Arabic adults consumed the following dietary supplements: 63.5% vitamin C, 60.1% vitamin D. The increased consumption of vitamin C may be attributed to its potent antioxidant and antiviral properties, and such evidence indicates it has a role in enhancing the promising immune response for combating the SARS-CoV-2 infection. Additionally, the fact that Vitamin D supplements in COVID-19 substantially improved clinical outcomes and had an antiviral effect suggests that they could be used to resolve or treat COVID-19 cases [43-45].

There are statistically significant differences between the COVID-19 case and control groups in terms of the total score for brain food consumption and junk food. This is aligned with the fact that dietary habits across various populations may contribute to the geographical variation in COVID-19 incidence. This variation is due to the entry of SARS-CoV-2 being mediated by transmembrane angiotensin-converting enzyme (ACE2). Dietary patterns are linked to ACE levels because blood ACE levels exhibit a rapid sensitivity to food intake, and consumption of specific foods may correlate with reduced mortality rates, e.g., The peptide LVLPGELAK in broccoli protein shows nearly double the ACE inhibitory activity, which correlates with a demonstrated hypotensive effect; a high-saturated fat diet elevates ACE, while certain plant foods exhibit ACE-inhibitory activity; processed foods high in fat, sugar, and salt can impair the immune system [46, 47]; and malnutrition, particularly regarding protein intake, may be a contributing factor. Energy, protein, and explicit micronutrient deficiencies and high prevalence of underweight and anemia are correlated with poor immune responses and augmented predisposition to diseases, and have recorded the highest incidence of COVID-19 [48].

### 4.2 Neurological Impairments Among the Studied Groups

The frequency of neurological disorders was higher in COVID-19 cases: 74 (42.5%) had smell impairments, 57 (32.3%) had cognitive disorders, and 27% had chronic fatigue syndrome. 44 (25.0%) had taste disorders, 34 (19.3%) had taste impairment, and 18 (10.3%) had hearing disorders compared to controls. However, the actual number was lower than the initial estimates [December 2021, SA]. The most frequently reported persistent symptoms were exhaustion (53.5%), loss of smell (35.0%), and loss of flavor (29.5%), in descending order. But the median in our analysis was nine months, although symptoms manifested four weeks after the condition's onset [21].

Having a SARS-CoV-2 infection significantly (p = 0.001) increases the odds of experiencing impairments in taste, appetite, smell, or hearing by approximately 30-, 33-, 3-, and 3-fold, respectively, compared to healthy controls. These effects were observed at 10 days (OR = 40.2, 95% CI = 2.204–733.2, *p* = 0.013) and 20 days (OR = 58.5, 95% CI = 3.278–1043.5, *p* = 0.005) post-infection. A multicenter study in Italy and Germany reported that one-quarter of patients experience persistent olfactory and gustatory impairments, whereas an Italian study found that SARS-CoV-2 did not result in long-term taste or smell deficits. In contrast, a German study challenged our findings, reporting that COVID-19 caused olfactory and gustatory abnormalities but only minimal hearing loss [15, 17, 32, 49].

The exact cause of post-COVID-19 neurological damage remains unclear; it could potentially stem from one of the following mechanisms: 2) Encephalitis occurs when a virus enters the central nervous system (CNS) through the axonal transmission of olfactory neurons, its direct ability to enter neurons and glial cells, and indirectly across the blood-brain barrier, resulting in neuronal damage (neuro-invasion) and dysfunction. 2) Hemorrhagic or ischemic strokes are the result of abnormalities in cerebral blood vessels and coagulation. 3) Extensive systemic inflammatory responses and peripheral organ dysfunction adversely affect the brain. Ischemia is a result of acute respiratory distress syndrome (ARDS) and breathing difficulties or their treatment [13, 17, 32, 40].

Regarding taste impairments, this analysis reported that salty, bitter, and “savory” (umami) flavors were most frequently affected, occurring in 25% of cases, and indicated that all tastes except sour, bitter, and the total score were impacted. Among the five taste types, the salty flavor was the most affected, consistent with previous research. In Germany, 20% of post-COVID-19 cases reported mild taste problems (12% for sweet, 8% for salty, 4% for bitter, and 2% for acidic). Multicenter studies in Italy and Germany found that only two cases (6.5%) experienced taste disturbances [15, 50].

Regarding chronic fatigue syndrome (CFS), the control group scored significantly lower on the Chronic Fatigue Syndrome (CFS) fatigue scale than COVID-19 subjects (*P* = 0.04). Many hypotheses link COVID-19 to chronic fatigue syndrome (CFS), including the idea that multiple organ damage caused by COVID-19 may induce CFS. 1) Inflammation, inflammatory mediators, and cell-mediated immunity may alter CFS-like conditions. 2) After COVID-19, debilitating symptoms, social isolation, and post-traumatic stress syndrome can cause depression and CFS. 3) Post-COVID-19 neurological symptoms include post-exercise malaise or exhaustion, sleeplessness and other sleep difficulties, headaches, loss of scent or taste, and memory or cognitive deficits. 4) Most survivors of COVID-19 experienced PCS, which included radiological changes and respiratory deterioration. About 30% of 30-59-year-olds with cognitive impairments reported severe work-related difficulties. This result is consistent with previous studies that have identified CFS as the most common persistent post-COVID-19 symptom, regardless of severity or respiratory distress [51].

### 4.3 Determinants And Frequency Of Post-COVID-19 Neurological Impairments Among Cases

In this face-to-face interview-based case-control study, the total cognitive score was significantly lower (*p* < 0.05) in the COVID-19 cases, with a mean±SD of 26.0 ± 3.76 and a range of 2–30. The decrease was attributed to differences in short-term memory, delayed recall, and orientation.

However, the classification of the cognitive score was insignificant, with 57 (33.2%) cases compared to 24 (26.1%) in the control group. This difference may be explained by the age range (16–80 years) and the fact that seven (7.6%) of the participants had a history of mental and cognitive impairment. In addition to the cognitive adverse effects of the post-COVID-19 vaccine, there was a risk of asymptomatic infection.

Cognitive impairment, which encompassed dysfunction in numerous cognitive domains, was prevalent following the COVID-19 pandemic. As per reports, an increasing number of recovered COVID-19 patients are experiencing neurological symptoms, including “slow thinking,” “difficulty focusing,” “confusion,” “lack of concentration,” “forgetfulness,” or “haziness in thought process.” These feelings of mental exhaustion can be conceptualized as “brain fog” and are associated with mild cognitive impairments [18].

This research demonstrated a substantial decrease in cognitive engagement, which encompassed attention, as well as a correlation between various domains. Almeria and his colleagues reported a substantial decline in both executive functions and attention, which is consistent with these findings. Roberta's research confirms the substantial delay in recall. It appears that patients who have recently contracted SARS-CoV-2 are experiencing challenges with memory, attention, executive function, and, particularly, speech fluency. This impairment is the result of structural damage, including ischemic or hypoxic injury to hippocampus, basal ganglia, or cerebellum lesions, as well as brain atrophy (particularly in the hippocampus) or disruption of functional connectivity, which are prevalent in ARDS survivors and may contribute to cognitive dysfunction [18, 52].

The pathogenic reasons *for cognitive impairment among post-COVID-19 cases* are unknown. However, the potential causes may include 1) cellular damage resulting from viral invasion. 2) secondary inflammatory responses and decreased ACE2 activity, which regulates neuroprotective and neuro-immunomodulatory functions, as well as certain pro-inflammatory cytokines that can cause mood and cognitive impairment. 3) Oxidative stress. 4) Hypoxia and ARDS (Reduced oxygen delivery to the brain may be a contributing factor, as two studies have reported a correlation between cognitive impairment and inferior pulmonary function) are prominent signs of COVID-19 that can damage many limbic areas, especially hippocampal CA3 neurons (CA2, CA3, and the dentate gyrus encode, while CA1 and the subiculum retrieve), which are involved in episodic memory and impair cognition. 5) Sepsis. Additionally, multi-organ damage caused by severe COVID-19 leads to endothelial dysfunction, hyperinflammation, autoimmunity, latent viral illness, multi-organ pathology, and autonomic nervous system dysfunction [18, 49, 53-55].

Regarding age, this analysis reported that COVID-19 cases with cognitive impairment were significantly older (44.6 ± 16.9 years, *p* < 0.001*) than cases without cognitive impairment, with an odds ratio of 1.04 (*p* = 0.04, 95% CI: 1.00–1.08). This finding is consistent with another study [52], which indicates that patients of all ages exhibit the most severe deficits in attention among various cognitive functions. Processing speed, verbal memory, and verbal fluency were the next domains in which the influence of age was evident. Interestingly, in the younger group, a larger proportion of patients exhibited altered scores, with a higher proportion corresponding to severe deficits. In the present investigation, age did not significantly affect post-COVID-19 cognitive impairment. Other studies have found significant associations with age, often in conjunction with other risk factors such as depression or preexisting mild cognitive impairment. Several studies have also linked age to post-COVID-19 outcomes due to lifestyle factors, including reduced physical activity [53, 54]. It is essential to consider the interaction between age and disease severity when interpreting these results, as COVID-19 severity is age-dependent, and age is an established risk factor for cognitive impairment in the general population [29, 56, 57].

Regarding sex, cognitive impairment was not significantly associated with the patient's sex, consistent with the findings reported by Heneghan AM in an earlier study. In contrast, research by Maggi G. found that women experienced more severe mental, cognitive, and psychological symptoms [27]. Townsend and colleagues reported that 67% of individuals experiencing post-viral fatigue were female [5]. Myalgic encephalomyelitis/chronic fatigue syndrome (ME/CFS) also shows a pronounced sex bias toward women [11]. In a study of Chinese patients from Wuhan who had been discharged from the hospital, 76% of patients with at least one persistent symptom six months after the onset of COVID-19 were female [58].

Regarding smoking, post-COVID-19 cases showed that smoking had a substantial impact on cognitive function. Smokers exhibited deficits in auditory-verbal and visuospatial learning, visuospatial memory, cognitive efficiency, executive function, general intelligence, and processing speed compared with nonsmokers [18, 59].

Regarding comorbidities, the findings suggest that neurological comorbidities, lower socioeconomic status, and advanced age may be risk factors for both long-term cognitive deficits and more severe COVID-19. A recent meta-analysis reported that neurological comorbidities were the most reliable predictors of cognitive impairment prior to COVID-19 onset. These factors are well-established risk factors for cognitive impairment and dementia in the general population. Interestingly, multivariable analyses showed no independent association between disease severity and cognitive outcomes.

Therefore, it is feasible that a SARS-CoV-2 infection could either exacerbate or reveal the extent of ongoing cognitive decline [57]. Furthermore, as previously mentioned, Beaud and his colleagues reported a pattern of cognitive deficits in 13 patients during the acute stage who had no history of cognitive, psychiatric, or neurological disorders. These deficits are like those found in other ARDS, so they linked them to COVID-19-related critical illness.

In terms of lifestyle factors, this study reports a significant association between post-COVID-19 cognitive impairments and higher consumption of junk food (8.6 ± 3.3, *P* = 0.04). Conversely, healthy behaviors such as a balanced diet, regular physical activity, and proper hygiene have been shown to enhance cognitive function, mental health, and overall well-being during COVID-19 [22]. This conclusion is consistent with a cohort study involving 10,775 individuals who were monitored over a median period of eight years. The study revealed that consumption of ultra-processed foods exceeding 19.9% of total daily caloric intake was associated with a more rapid decline in global cognitive performance and executive function compared with consumption below this threshold. The proportion of daily energy derived from ultra-processed foods was linked to cognitive decline in participants under 60 years, highlighting the need for preventive interventions in middle-aged adults. Furthermore, this association was observed in individuals with a low healthy diet score, whereas no such link was found in those with a high healthy diet score [60, 61].

The total Montreal Cognitive Assessment score was moderately and significantly positively correlated with other neurological symptoms in critically ill patients, including impairments in hearing, taste, smell, or appetite. The results indicated that cognitive impairment was more prevalent in this population. This finding may be explained by the concept of cognitive reserve (CR), which moderates pathology and clinical outcomes by allowing the brain to actively compensate for damage through preexisting cognitive processing or compensatory strategies. Evidence suggests that individuals with a high CR are better able to manage brain injury than those with a low CR. Therefore, we hypothesize that CR contributes to the relationship between neurocognitive outcomes and medical risk factors for brain injury, consistent with prior research [53, 62].

### 4.4 Strengths and Limitations

The present investigation is a multinational study that matched a sample of recovered COVID-19 patients with healthy, well-matched controls from three different countries. Data were collected through face-to-face interviews conducted by well-trained physicians, who ensured proper recruitment of cases and addressed any discrepancies, and the assessment tools were validated. The study examined how five neurological deficits, including taste, smell, hearing, appetite, and fatigability, are associated with various other factors, including physical activity, consumption of brain-healthy foods, and intake of eight nutritional supplements. Multiple validated instruments were used to assess the medium- to long-term consequences of SARS-CoV-2 infection.

This case-control study has several limitations. Examining post-COVID-19 neurological impairments is challenging due to the emergence of the delta variant and other strains, which may have evolved after vaccination. The duration of illness following the last dose or type of vaccination remains unexplored, and temporal data on the progression of symptoms were not collected. Additionally, the health status of individuals prior to vaccination was not examined. In a retrospective exposure analysis, it is difficult to separate COVID-19-related effects from other contemporary factors, meaning causality cannot be established. Although the questionnaire was validated in three countries, potential cultural differences or linguistic biases may have influenced the reporting and interpretation of symptoms. The onset and persistence of cognitive symptoms after infection are also important but were not fully captured. Despite these limitations, our results are consistent with findings from other global studies. Furthermore, the use of both objective and subjective activity measures, along with self-reported data on healthy and unhealthy eating, may introduce additional biases. For example, while the association between junk food consumption and cognitive impairment is intriguing, the causal relationship remains unclear.

## CONCLUSION

Cognitive dysfunction, olfactory, gustatory, and auditory abnormalities, appetite disturbances, and chronic fatigue were more common in recovered COVID-19 patients than in matched controls, highlighting the substantial burden of post-COVID-19 neurological deficits. Factors such as smoking, poor dietary habits, unemployment, female sex, and advanced age were significantly associated with cognitive impairment. These findings underscore the need for targeted interventions, including cognitive rehabilitation and lifestyle modifications, to mitigate long-term neurological effects. Further research is required to elucidate the underlying mechanisms and to develop effective management strategies for post-COVID-19 cognitive impairment.

## RECOMMENDATIONS

- COVID-19 patients should continue to receive monitoring to track and treat their post-COVID issues even after they have recovered.
- Health education concerning post-COVID-19 symptoms, such as neurological damage, is crucial, particularly for at-risk populations, such as the elderly, women, the unemployed, smokers, and those with comorbidities. Therefore, to prevent potential complications, it is important to discuss scheduling follow-ups for early diagnosis, evaluation, and treatment of the neurological impairment.
- It is advised to investigate the possibility of virtual examinations for determining neurological abnormalities in post-COVID-19 patients, given the difficulties in setting up follow-up sessions.
- To support the rehabilitation of neurological functioning in people afflicted by COVID-19, it is recommended to promote a healthy lifestyle that includes regular physical activity, proper supplements, and a reduction in the consumption of unhealthy brain food.
- Comprehensive post-COVID-19 rehabilitation programs ought to be offered both during and after the patient's hospital stay.
- More prospective research is required to better understand post-COVID-19 neurological deficits, their long-term implications, and the best therapeutic strategies.
- It’s recommended to validate such cognitive impairment tools that take potential cultural or linguistic biases into account.

There is a need for preventive interventions targeting cognitive impairments in middle-aged adults, particularly focusing on reducing the consumption of unhealthy ultra-processed foods and increasing the intake of brain-healthy foods.

## AUTHOR’S CONTRIBUTIONS

The authors’ contributions are as follows. Conceptualization was carried out by S.A.A. and Y.A. Methodology was developed by S.A., E.F.A., Y.A., I.F.D., and E.M. Validation was performed by M.T.H., N.H., and Y.A. Formal analysis was conducted by S.A. and N.H., while data curation was handled by I.D., E.M., Y.A., and E.F.A. The original draft was prepared by S.A. and M.M., and review and editing were completed by M.H., N.H., E.A., M.E., and C.S.L. Visualization was carried out by SA, LS, EF, and M.T.H. Supervision was provided by S.A., J.S., Y.A. and C.S.L, with project administration led by M.T.H. and S.S. All authors have read and agreed to the published version of the manuscript.

## Figures and Tables

**Fig. (1):**
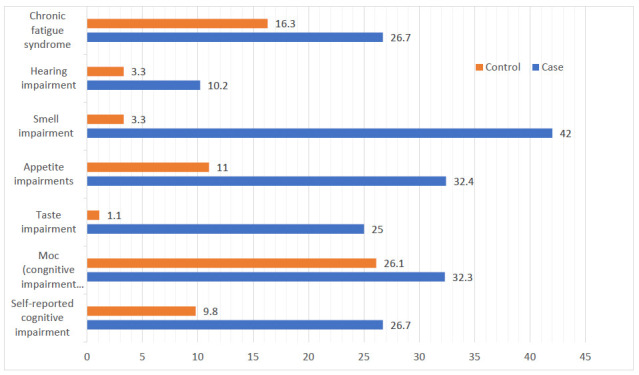
Bar charts show the percentages of neurological impairments among the studied groups.

**Table 1:** Demographic and lifestyle determinants among the studied groups.

	**Case** **T= 176** **F (%)**	**Control** **T= 92** **F (%)**	*P*
**The demographical determinants**			
**Age (years)** Mean ± SD Range	36.4 ± 15.3 15-80	36.1±13.6 16-80	T= 1.2. (0.25)
**Sex** Male Female	53 (30.1) 123 (69.1)	31 (33.7) 61 (66.3)	0.58
**Level of education** Illiterate Primary /secondary High school /university Postgraduate	4 (2.3) 39 (22.2) 122 (69.3) 11 (6.3)	3 (3.3) 18 (19.6) 68 (73.9) 3 (3.3)	0.64
**Residency** Rural Urban	29 (16.5) 147 (83.5)	22 (23.9) 70 (76.1)	0.19
**BMI (kg/m^2^)** Mean ± SD	33.7 ± 6.1	33.6 ± 7.8	T= 1.0 (0.99)
**Occupation** Students Working in jobs requires mental skills Other jobs Retired	36 (20.5) 45 (25.6) 30 (17.0) 65 (36.9)	19 (20.7) 18 (19.6) 24 (26.1) 31 (33.7)	0.32
**Marital status** Divorced / widow Married Single	11 (6.2) 114 (64.8) 51 (29.0)	3 (3.3) 57 (62.0) 32 (34.8)	0.58
**Family History** Auto-immune disease Consanguinity Diabetes Mellitus(DM) Neurological illness (CNS)(Alzheimer) Mental psychiatric illness No family history	16 (9.1) 14 (8.0) 1 (0.6) 11 (6.3) 14 (8.0) 120 (68.2)	10 (10.9) 4 (4.3) 0 (0.0) 2 (2.2) 5 (5.4) 71 (77.2)	0.39
**The lifestyle determinants**			
S**mokers** No Ex-smoker Passive smokers or smokers Shisha smokers	99 (56.3) 12 (6.8) 49 (27.8) 16 (9.1)	47 (51.1) 16 (17.4) 27 (29.3) 2 (2.2)	0.011*
**Brain’s healthy food consumption total score** Mean ± SD	12.0 ± 4.02	14.2 ± 3.7	4.23 (0.00*)
**Brain’s unhealthy food consumption total score** Mean ± SD	10.1 ± 2.8	7.4 ± 3.4	6.67 (0.00*)
**The consumption of Dietary Supplements**	95 (53.9)	43 (46.7)	1.27 (0.23)
**Dietary Supplements consumption** Vitamin B12 Folic Acid Iron Multi- vitamina Vitamin B/C/D/E Omega 3 Zinc Collagen	T= 95 14 (14.7) 28 (29.5) 24 (25.3) 19 (20.0) 32 (33.7) 15 (15.8) 25 (26.3) 3 (3.2)	T= 43 7 (16.3) 13 (30.2) 14 (32.6) 12 (27.9) 5 (11.6) 12 (27.9) 9 (20.9) 2 (4.7)	0.88 0.91 0.79 0.34 0.01* 0.09 0.85 0.91
**Level of Physical activity steps/day** Sedentary (<5000) Low (5000-<7000) Average (7000-10,000) High (more than 10,000)	49 (27.8) 60 (34.1) 46 (26.1) 21 (11.9)	16 (17.4) 36 (39.1) 36 (39.1) 4 (4.3)	10.2 (0.02*)

*P<0.05. There was a statistically significant difference.

**Table 2:** The current COVID-19 vaccination status and the history of SARS-CoV-2 infection among the studied groups.

	**Case** **T= 176** **F (%)**	**Control** **T= 92** **F (%)**	**Test** **(*P*)**
**The COVID-19 vaccination history**			
**The current COVID-19 vaccination status (doses)** Un vaccinated Single doses Two doses Three doses	100 (56.8) 16 (9.1) 48 (27.3) 12 (6.8)	58 (63.0) 10 (10.9) 24 (26.1) 0 (0.0)	6.89 (0.07)
**Adverse effects after COVID-19 vaccine** General Systemic Local No	T= 76 4 (5.3) 24 (31.6) 41 (53.9) 6 (7.9)	T= 36 2 (5.6) 3 (8.3) 22 (61.1) 9 (25.0)	8.04 (0.03*)
**The history of SARS-CoV-2 infection**			
**COVID-19 cases**	176 (100.0)	0 (0.0)	-
**Time passed (m)** Median (mean ± SD) Range	9 (8.3±6.1) 2-26 m	-	-
**Resolution of COVID-19 symptoms** Asymptomatic Resolved Unresolved	29 (16.5) 145 (82.4) 2 (1.1)	-	-
**Management** Home Hospital ICU	152 (86.4) 15 (8.5) 9 (5.1)	-	-

*P<0.05. There was a statistically significant difference / ICU= intensive care unit/ m= months.

**Table 3:** Neurological assessment among the studied groups.

	**Case** **T= 176** Mean ± SD Range	**Control** **T= 92** Mean ± SD Range	**Test** **(*p*)**
The Montreal Cognitive Assessment (MoCA)			
**Animal naming**	2.8 ± 0.3 1-3	2.8 ± 0.4 1-3	T= 0.37 p= (0.72)
**Executive function**/**visuospatial ability**	4.2 ± 1.1 0-5	4.1 ± 1.3 0-5	1.08 p= (0.27)
**Orientation**	4.6 ± 1.4 1-6	5.2 ± 1.1 1-6	T= 4.02 p= (0.00*)
**Language**	2.59 ± 0.6 0-3	2.65 ± 0.5 1-3	T= 0.91 (0.37)
**Abstraction**	1.73 ± 0.6 0-2	1.72 ± 0.56 0-2	T= 0.20 (0.84)
**Short-term memory**/delayed recall	3.7 ± 1.1 0-5	4.12 ± 0.9 2-5	T= 2.82 (0.01*)
**Attention**	5.81 ± 0.41 4-6	5.88 ± 0.33 5-6	T= 1.39 (0.17)
**Total cognitive score**	26.0 ± 3.76 2-30	27.9 ± 2.98 15-30	T= 6.1 (0.00*)
**The type of taste disturbance** Sweet Salty Sour Bitter & ‘savory’ (umami) tastes.	T= 44 7 (15.9) 18 (40.9) 1 (2.3) 18 (40.9)	T= 1 0 (0.0) 1 (1.1) 0 (0.0) 0 (0.0)	<0.001*
**The SARS-CoV-2 infection risk assessment and the following post-COVID-19 neurological impairment**			
	**OR (95% Confidence Interval)**		***P***
Taste disturbance	30.33 (4.11-224.1)		0.00*
Appetite disturbance	3.5 (1.74-7.14)		0.00*
Smell disturbance	32.6 (7.79-136.8)		0.00*
Hearing disturbance	3.38 (0.97-11.8)		0.00*
Cognitive impairment	1.7 (1.3-6.1)		0.00*

*P<0.05. There was a statistical significant difference.

**Table 4:** The association between post-COVID-19 cognitive impairment and demographics and the life style determinants among the studied groups.

-	**Post-COVID-19 cognitive impairment**		
-	**Yes** **T= 57** **F (%)**	**No** **T= 119** **F (%)**	**Test** ***(P)***
**a) The demographic determinants**			
**Age(y)** mean ± SD Range	44.6 ± 16.9 16-77	36.2 ± 13.8 16-80	3.49 (<0.001*)
**Sex** Male Female	17 (16.7) 85 (83.3)	36 (48.6) 38 (48.6)	12.7 (<0.001*)
**Level of education** Illiterate Primary/ Secondary High school /university Postgraduate	2 (2.0) 19 (18.6) 78 (76.5) 3 (2.9)	2 (2.7) 20 (27.0) 44 (59.5) 19 (18.6)	16.1 (<0.001*)
**Residency** Rural Urban	14 (13.7) 88 (86.3)	15 (20.3) 59 (79.7)	2.45 (0.13)
**Weight (kg)** Mean ± SD Range	77.4 ± 16.7 48-120	74.5 ± 15.8 41-120	1.12 (0.13)
**Occupation** Students Working in jobs requires mental skills Other jobs Retired	26 (25.5) 18 (17.6) 16 (15.7) 42(41.2)	10 (13.5) 27 (36.5) 14 (18.9) 23 (31.1)	23.9 (<0.001*)
**Marital status** Divorced / widow Married Single	2 (2.0) 65 (63.7) **35 (34.3)**	9 (12.2) 49 (66.2) 16 (21.6)	8.41 (0.04*)
**Family History** Auto-immune disease Consanguinity Diabetes Mellitus(DM) Neurological illness (CNS)(Alzheimer) Mental psychiatric illness No family history	5 (8.8) 3 (5.3) 0 (0.0) 3 (5.3) 6 (10.5) 40 (70.2)	11 (9.2) 11 (9.2) 1 (0.8) 8 (6.7) 8 (6.7)) 80 (68.2)	2.14 (0.83)
b) **The life style determinants**			
S**mokers** No Ex-smoker Passive smokers Shisha smokers	33 (57.9) 1 (1.8) 21 (36.8) 2 (3.5)	66 (55.5) 11 (9.2) 28 (23.5) 14 (11.8)	8.56 (0.04*)
**Brain’s-healthy food consumption total score** Mean ± SD Range	11.9 ± 4.1 4-19	12.0 ± 4.1 1-20	0.117 (0.45)
**Brain’s unhealthy food consumption total score** Median (mean ± SD) Range	9 (8.6 ± 3.3) 0-14	7 (6.8 ± 3.6) 0-16	2.3 (0.04*)
**Physical activity (steps per day)** Sedentary (<5000) Low (5000-<7000) Average (7000-10,000) High (more than 10,000)	19 (33.3) 21 (36.8) 15 (26.3) 2 (3.5)	30 (25.2) 39 (32.8) 31 (26.1) 19 (16.0)	6.11 (0.11)
**Self-reported cognitive impairment** Yes No	47 (82. 5) 10 (17.5)	10 (8.5) 109 (91.6)	96.7 (<0.0001*)

*P<0.05 there was a statistical significant difference.

**Table 5:** Factors associated with the post-COVID-19 cognitive impairment.

**post-COVID-19 cognitive impairment**		***P***	**OR**	**95% Confidence Interval for Exp (B)**	
				Lower Bound	Upper Bound
Intercept		.999	-	-	-
Age (y)		.047*	1.040	1.001	1.082
Sex	Female	.316	2.005	.514	7.814
	Male (reference)	.	.	.	.
Level of education	High, university	.229	.552	.210	1.454
	Illiterate	.421	3.297	.181	60.117
	Post gradate	.	1.297E-9	1.297E-9	1.297E-9
	Primary, Secondary(reference)	.	.	.	.
Residence	Rural	.620	1.294	.468	3.579
	Urban (reference)	.	.	.	.
Occupation	Other jobs	.923	1.067	.283	4.020
	Retired	.323	1.836	.550	6.127
	Student	.573	1.532	.347	6.766
	Working in a job requires mental skill reference).				
Marital state
	Married	.999	8.407E-9	.000	.^c^
	Single	.999	7.299E-9	.000	.^c^
	Divorced /widow (reference)	.	.	.	.
Smoking	Ex-smoker	.999	1.499E-9	.000	.^c^
	No	.625	1.638	.226	11.865
	Passive smoker	.440	2.268	.284	18.112
	Shisha smoker(reference)	.	.	.	.

**Table 6:** The association between post-COVID-19 cognitive impairment, the history of SARS-CoV-2 infection, the current vaccination status, and the associated neurological impairment.

	**Post-COVID-19 cognitive impairment**			
	**Yes** **T= 57** **F (%)**		**No** **T= 119** **F (%)**	**Test X^2^** **(*P*)**
**The current status of COVID-19 vaccination**				
**The current status of COVID-19 vaccination (doses)** Not vaccinated Single doses Two doses Three doses	38 (66.7) 4 (7.0) 14 (24.6) 1 (1.8)		62 (52.1) 12 (10.1) 34 (28.6) 11 (9.2)	5.23 (0.16)
**AEs after the COVID-19 vaccine** General Systemic Local No	0 (0.0) 6 (10.5 11 (19.2) 40 (70.1)		4 (3.4) 18 (15.1) 30 (25.2) 67 (56.3)	4.3 (0.23)
**The history of SARS-CoV-2 infection**				
**SARS-CoV-2 infection** Before vaccination After the 1^st^ doses After the 2^nd^ doses After the 3^rd^ doses	49 (86.0) 4 (7.0) 4 (7.0) 0 (0.0)		82 (68.9) 18 (15.1) 16 (13.4) 3 (2.5)	6.37 (0.09)
**The self-reported resolution of COVID-19 symptoms** Asymptomatic Resolved Unresolved	8 (14.0) 48 (84.2) 1 (1.8)		20 (16.8) 98 (82.2) 1 (0.8)	0.98 (0.81)
**Management** Home Hospital ICU	47 (82.5) 6 (10.5) 4 (7.0)		105 (88.2) 9 (7.6) 5 (4.2)	1.14 (0.56)
**Associated neurological impairments**				
**Associated neurological impairments** Hearing impairments Appetite impairments Taste impairments Smell impairments	9 (15.8) 15 (26.3) 14 (24.5) 20 (35.1)		9 (7.6) 42 (35.3) 30 (25.2) 54 (45.4)	2.8(0.11) 1.42(0.30) 1.45(0.31) 1.68(0.25)
**The correlation between the total Montreal cognitive score and the presence of other neurological symptoms**	r(*p*) 0.34 (0.02*)		r(*p*) 0.19 (0.04*)	-

*P<0.05 there was a statistically significant difference/ X^2^ chi-square test /r Pearson's correlation coefficient.

## Data Availability

The data sets used and/or analyzed during the current study are not publicly available for legal and ethical reasons. Further inquiries can be directed to the corresponding author. On reasonable request, the corresponding author can provide the datasets.

## LIST OF ABBREVIATIONS

ARDS: Acute Respiratory Distress Dyndrome
ACE2: Angiotensin-converting Enzyme
BMI: Body Mass Index
CDC: Centers for Disease Control and Prevention
CSF: Cerebrospinal Fluid
CFSLWIFS: CFS-like with Insufficient Fatigue Syndrome
CFS: Chronic Fatigue Syndrome
CR: Cognitive Reserve
CI: Confidence Interval
COVID-19: Coronavirus Disease 2019
DM: Diabetes Mellitus
MCI: Mild Cognitive Impairment
MoCA: Montreal Cognitive Assessment
ME/CFS: Myalgic Encephalomyelitis/Chronic Fatigue Syndrome
PCR: Polymerase Chain Reaction
PCS: Post-acute COVID-19 Syndrome
SARS-CoV2: Severe Acute Respiratory Syndrome Coronavirus 2
SEs: Side Effects
aOR: The Adjusted Odds Ratios
BBB: The Blood-brain Barrier
CNS: The Central Nervous System
WHO: The World Health Organization
TDI Test: Threshold Discrimination, and Identification Test

## STANDARDS OF REPORTING

STROBE guidelines were followed.
