# Supplementary material for: Post-COVID-19 Cognitive Dysfunction: Analyzing the Role of Age, Lifestyle, and Neurological Impairments: A Multi-Centric Case-Control Study
Source: Clin Pract Epidemiol Ment Health. 2025 Oct 31;21:e17450179395261. doi: 10.2174/0117450179395261251006055231 (PMC12895434; doi:10.2174/0117450179395261251006055231)
Supplement: Supplementary file 1 — Supplementary material is available on the Publisher’s website along with the published article. [file CPEMH-21-E17450179395261_SD1.pdf]

**Clin. Pract. Epidemiol. Ment. Health**

ISSN: 1745-0179

*DOI: 10.2174/0117450179395261251006055231, 2025, 21, e17450179395261*



**SUPPLEMENTARY MATERIAL** **OPEN ACCESS**

# Post-COVID-19 Cognitive Dysfunction: Analyzing the Role of Age, Lifestyle, and Neurological Impairments: A Multi-Centric Case-Control Study

Samar A. Amer[1,*], Ines F. Djelleb[2], Ehab M. Ishteiwy[3], Mostafa Meshref[4], Youmna A. Amer[5], Jaffer Shah[6], Mahmoud Tarek Hefnawy[7,*], Noha A. Hashim[8], Carlos Schönfeldt-Lecuona[9], Mohamed E.G. Elsayed[9,10] and Eman F. Ali[11]

[1]*Department of Public Health and Community Medicine, Faculty of Medicine, Zagazig University, Zagazig, Egypt*
[2]*Faculty of Medicine, Badji Mokhtar University, Annaba 23000, Algeria*
[3]*Albayda Medical Centre (AMC), Omar-Almukhtar University, Albayad, Libya*
[4]*Neurology Department, Faculty of Medicine, Alazhar University, Cairo, Egypt*
[5]*Department of Rheumatology and Rehabilitation, Faculty of Medicine, Zagazig University, Zagazig, Egypt*
[6]*Medical Research Centre, Kateb University, Kabul, Afghanistan*
[7]*Faculty of Medicine, Zagazig University, Zagazig, Egypt*
[8]*Neurology Department, Faculty of Medicine, Zagazig University, Zagazig, Egypt*
[9]*Department of Psychiatry and Psychotherapy III, University of Ulm, Leimgrubenweg 12-14, 89075 Ulm, Germany*
[10]*Department of Psychiatry, School of Medicine and Health Science, Carl von Ossietzky University Oldenburg, Oldenburg, Germany*
[11]*Department of Psychiatry, Faculty of Medicine, Zagazig University, Zagazig, Egypt*

© 2025 The Author(s). Published by Bentham Open.
This is an open access article distributed under the terms of the Creative Commons Attribution 4.0 International Public License (CC-BY 4.0), a copy of which is available at: https://creativecommons.org/licenses/by/4.0/legalcode. This license permits unrestricted use, distribution, and reproduction in any medium, provided the original author and source are credited.

*Address correspondence to these author at the Department of Public Health and Community Medicine, Faculty of Medicine, Zagazig University, Egypt and Faculty of Medicine, Zagazig University, Egypt; Medical Research Group of Egypt (MRGE), Cairo, Egypt; E-mails: mahmoudhefnawy77@gmail.com, dr_samar11@yahoo.com

*Cite as:* Amer S, Djelleb I, Ishteiwy E, Meshref M, Amer Y, Shah J, Hefnawy M, Hashim N, Schönfeldt-Lecuona C, Elsayed M, Ali E. Post-COVID-19 Cognitive Dysfunction: Analyzing the Role of Age, Lifestyle, and Neurological Impairments: A Multi-Centric Case-Control Study. Clin Pract Epidemiol Ment Health, 2025; 21: e17450179395261.
http://dx.doi.org/10.2174/0117450179395261251006055231

Published: October 31, 2025

***Send Orders for Reprints to reprints@benthamscience.net***

**Table A. The consumption of brain food diet (Healthy and unhealthy).**

| | **Case (Post-COVID-19) T= 176 F (%)** | **Control T= 92 F (%)** | **Test (p)** |
|---|---|---|---|
| Omega 3 (nuts, fish, and tuna....) | **99 (56.3)** | **14 (15.2)** | **6.7** |
| Never | **15 (8.5)** | **14 (15.2)** | **(0.00*)** |
| Rare | **33 (18.8)** | **27 (29.3)** | |
| sometimes | **22 (12.5)** | **22 (23.9)** | |
| Often | **7 (4.0)** | **15 (16.3)** | |
| Always | | | |

*(Table A.) contd.....*

| | Case (Post-COVID-19) T= 176 F (%) | Control T= 92 F (%) | Test (p) |
|---|---|---|---|
| Vitamin D (Sun exposure of more than 1h)<br>Never<br>Rare<br>sometimes<br>Often<br>Always | **36 (20.5)**<br>**22 (12.5)**<br>**46 (26.1)**<br>**29 (16.5)**<br>**43 (24.4)** | **6 (6.5)**<br>**9 (9.8)**<br>**35 (38.0)**<br>**16 (17.4)**<br>**26 (28.3)** | **2.23**<br>**(0.39)** |
| More than two letters of water<br>Never<br>Rare<br>sometimes<br>Often<br>Always | **38 (21.6)**<br>**22 (12.5)**<br>**15 (8.5)**<br>**23 (13.1)**<br>**78 (44.3)** | **5 (5.4)**<br>**17 (18.5)**<br>**18 (19.6)**<br>**23 (25.0)**<br>**29 (31.5)** | |
| Strawberry or grab<br>Never<br>Rare<br>sometimes<br>Often<br>Always | **8 (4.5)**<br>**33 (18.8)**<br>**70 (39.8)**<br>**30 (17.0)**<br>**35-(19.9)** | **6 (6.5)**<br>**18 (19.6)**<br>**28 (30.4)**<br>**34-(37.0)**<br>**6-(6.5)** | **0.82**<br>**(0.41)** |
| Leafy Green vegetables<br>Never<br>Rare<br>sometimes<br>Often<br>Always | **5-(2.8)**<br>**20 (11.4)**<br>**35 (19.9)**<br>**55 (31.3)**<br>**61 (34.7)** | **3 (3.3)**<br>**9 (9.8)**<br>**15 (16.3)**<br>**35 (38.0)**<br>**30 (32.6)** | **0.24 (0.81)** |
| Caffeine (cup of coffee or more than 2 cup of tea)<br>Never<br>Rare<br>sometimes<br>Often<br>Always | **64 (36.4)**<br>**7 (4.0)**<br>**16 (9.1)**<br>**11 (6.3)**<br>**78 (44.3)** | **7 (7.6)**<br>**4 (4.3)**<br>**9 (9.8)**<br>**12 (13.0)**<br>**60 (65.0)** | **4.98 (0.00*)** |
| Vegetable oil (Oliver oil,)<br>Never<br>Rare<br>sometimes<br>Often<br>Always | **67 (38.1)**<br>**43 (24.4)**<br>**31 (17.6)**<br>**13 (7.4)**<br>**22 (12.5)** | **23 (25.0)**<br>**11 (12.0)**<br>**24 (26.1)**<br>**19 (207)**<br>**15 (16.3)** | **3.33 (0.01*)** |
| Total score | **12.0 ± 4.1** | **14.1 ± 3.7** | **0.00*** |

## The English Version of the Questionnaire

### 1- Informed consent

### 2- Demographic

- Sex (male, female)
- Residence (urban, rural)
- Educational level
- Nationality (Saudi, non-Saudi)
- Occupation
- Age, in years
- Body mass index (BMI); body weight (kg)/ (height in meter )2
- Marital status (single, widow, married, divorced)
- Comorbidities (non, mental and psychological, neurological disorder, autoimmune diseases, Diabetes mellitus, cardiovascular diseases, others)

### 3- History of COVID-19 infection

- Since (duration in months)
- Before the vaccination, after the 1$^{st}$ dose, after the 2$^{nd}$ dose, after the 3$^{rd}$ dose
- Management plan: (Asymptomatic, at home, required hospital admission, required ICU admission, required mechanical ventilation)

● Resolved symptoms (yes, no)

**4- COVID-19 vaccination history (Event data) per each dose; (1st, 2nd, and the 3rd dose)**

● Types (AstraZeneca, Johnson and Johnson (J&J), Sputnik light, Sepotic -7 ,Sinopharm , Sinovac ,Moderna , Pfizer ,mixed ) vaccines

● Adverse Effects;

i. No side effects (SE

ii. Local (injection site) SE e.g., pain, heaviness, redness and swelling.

iii. General SE

iv. Systemic SE

v. Serious SE

**5- Nutritional, and supplement history**

| Item | Not at all | Less than three times a month | Once or twice a week | 3-5 times a week | more than 5 times a week |
|---|---|---|---|---|---|
| Omega-3, Tuna fishe, Nuts | - | - | - | - | - |
| Vit D, Sun e | - | - | - | - | - |
| Vitamin D or prolonged exposure of long bones (more than an hour) to direct sunlight | - | - | - | - | - |
| Processed meats such as hamburgers and hot dogs | - | - | - | - | - |
| Fried foods | - | - | - | - | - |
| Sugar in excess of three teaspoons | - | - | - | - | - |
| Drinking more than two liters of water per day | - | - | - | - | - |
| Berries or grapes | - | - | - | - | - |

**6- Exercise level**

| Level | Description |
|---|---|
| Sedentary lifestyle | Less than 5,000 steps per day and less than 2.5 hours of physical activity per week. |
| Low activity level | 5,000 to less than 7,500 steps per day or 2.5 to less than 5 hours of physical activity per week. |
| Moderate activity level | 7,500 to 10,000 steps per day or 2.5 to less than 5 hours of physical activity per week. |
| High activity level | More than 10,000 steps per day or more than 5 hours of physical activity per week. |

**7- Sleep**

● Average duration in hours

● Continuity ( continuous, interrupted)

● Quality of sleep (very bad, poor, accepted, good, very good)

**8- Screen use**

● Average duration in hours

**9- Supplement Intake**

● No at all

● Zinc

● Iron

● Vitamin A

● Selenium

● Vitamin D

● Vitamin E

● Vitamin C

● Multi vitamin

● Collagen

● Vitamin B12

● Others ( Mention)

**10. Montreal Cognitive Assessment Test (MOCA test)**

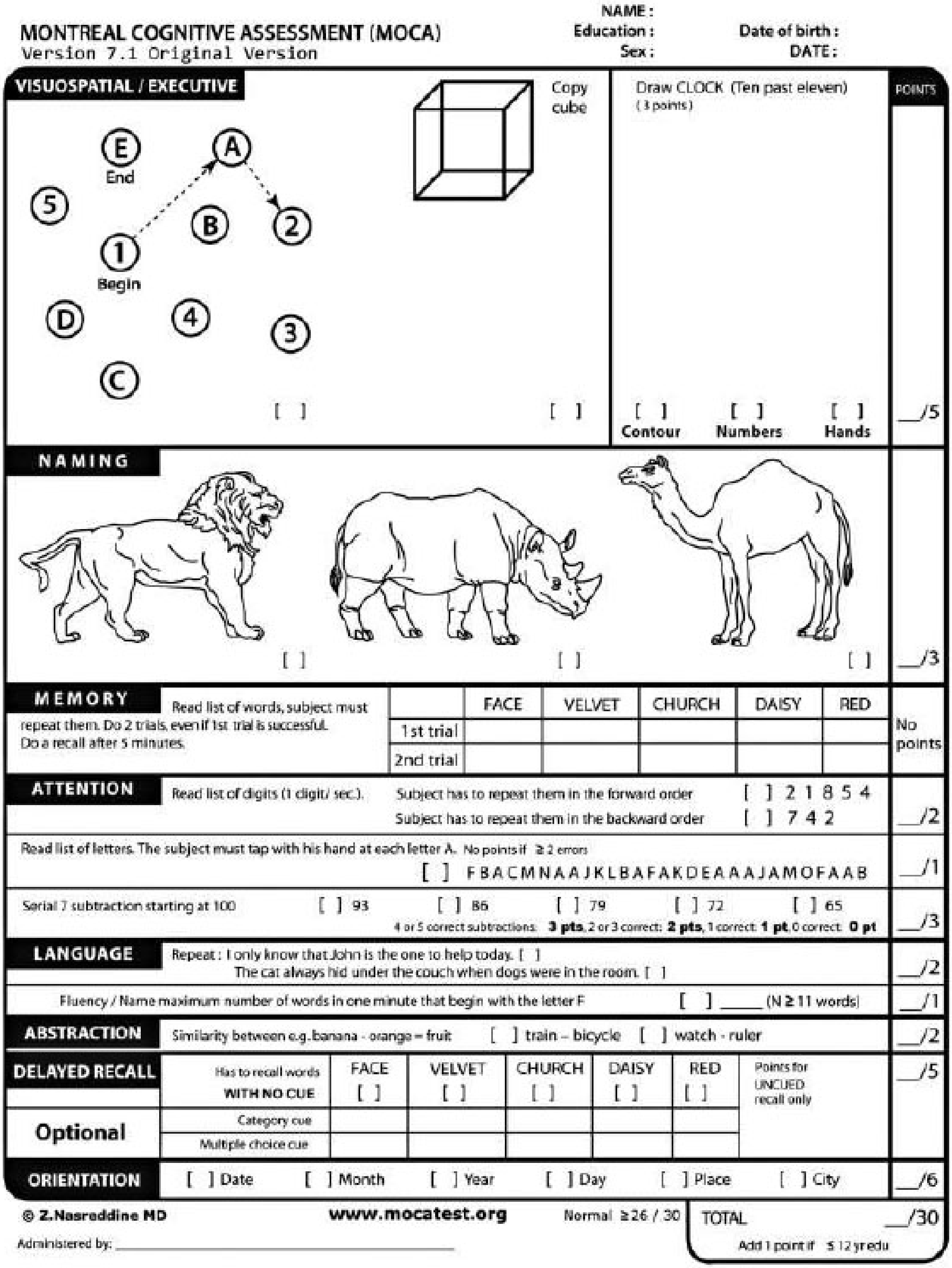

MONTREAL COGNITIVE ASSESSMENT (MOCA)
Version 7.1 Original Version

NAME :
Education : Date of birth :
Sex : DATE :

VISUOSPATIAL / EXECUTIVE — POINTS



[ ] Copy cube [ ] Draw CLOCK (Ten past eleven) (3 points) [ ] Contour [ ] Numbers [ ] Hands ___/5

NAMING [ ] [ ] [ ] ___/3

| MEMORY Read list of words, subject must repeat them. Do 2 trials, even if 1st trial is successful. Do a recall after 5 minutes. | | FACE | VELVET | CHURCH | DAISY | RED | No points |
|---|---|---|---|---|---|---|---|
| | 1st trial | | | | | | |
| | 2nd trial | | | | | | |

ATTENTION Read list of digits (1 digit/ sec.). Subject has to repeat them in the forward order [ ] 2 1 8 5 4
Subject has to repeat them in the backward order [ ] 7 4 2 ___/2

Read list of letters. The subject must tap with his hand at each letter A. No points if ≥ 2 errors
[ ] F B A C M N A A J K L B A F A K D E A A A J A M O F A A B ___/1

Serial 7 subtraction starting at 100 [ ] 93 [ ] 86 [ ] 79 [ ] 72 [ ] 65
4 or 5 correct subtractions: **3 pts**, 2 or 3 correct: **2 pts**, 1 correct: **1 pt**, 0 correct: **0 pt** ___/3

LANGUAGE Repeat : I only know that John is the one to help today. [ ]
The cat always hid under the couch when dogs were in the room. [ ] ___/2

Fluency / Name maximum number of words in one minute that begin with the letter F [ ] ____ (N ≥ 11 words) ___/1

ABSTRACTION Similarity between e.g. banana - orange = fruit [ ] train – bicycle [ ] watch - ruler ___/2

| DELAYED RECALL | Has to recall words WITH NO CUE | FACE [ ] | VELVET [ ] | CHURCH [ ] | DAISY [ ] | RED [ ] | Points for UNCUED recall only | ___/5 |
|---|---|---|---|---|---|---|---|---|
| Optional | Category cue | | | | | | | |
| | Multiple choice cue | | | | | | | |

ORIENTATION [ ] Date [ ] Month [ ] Year [ ] Day [ ] Place [ ] City ___/6

© Z.Nasreddine MD www.mocatest.org Normal ≥26 / 30 TOTAL ___/30
Add 1 point if ≤ 12 yr edu
Administered by: ____________________

**11. Chronic Fatigue Symptoms (CFS) Questionnaire**

CFS Questionnaire Instructions

Score each symptom in 2 steps. First, has the symptom caused you problems on more than half of the days in the past 6 months? Second, what was the overall severity of the symptom for the past 6 months?

| Symptom | Is this a problem more than half the time? | | Severity in the past 6 months | | | | | Score |
|---|---|---|---|---|---|---|---|---|
| | Yes | No | None | Trivial | Mild | Moderate | Severe | |
| Fatigue | Yes | No | 0 | 1 | 2 | 3 | 4 | |
| Short term problems with memory or concentrating | Yes | No | 0 | 1 | 2 | 3 | 4 | Sum8 |
| Sore throat | Yes | No | 0 | 1 | 2 | 3 | 4 | |
| Sore lymph nodes (neck, armpits, groin) | Yes | No | 0 | 1 | 2 | 3 | 4 | |
| Muscle pain | Yes | No | 0 | 1 | 2 | 3 | 4 | |
| Joint pain | Yes | No | 0 | 1 | 2 | 3 | 4 | |
| Headaches | Yes | No | 0 | 1 | 2 | 3 | 4 | |
| Difficulty sleeping or unrefreshing sleep | Yes | No | 0 | 1 | 2 | 3 | 4 | |
| Extreme fatigue after exercise or mild exertion | Yes | No | 0 | 1 | 2 | 3 | 4 | |

| | | Fatigue | |
|---|---|---|---|
| | | None, Trivial, Mild | Moderate, Severe |
| Sum of ancillary criteria (Sum8) | 0–13 | ☐ Normal | ☐ Chronic idiopathic fatigue (CIF) |
| | 14–32 | ☐ CFS-like with insufficient fatigue syndrome (CFSLWIFS) | ☐ Chronic fatigue syndrome (CFS) |

| | |
|---|---|
| What would you do if you were better tomorrow? | ☐ 1. I have a list of things to do<br>☐ 2. I would stop being sad<br>☐ 3. Not sure |
| What happens if you walk a long distance? | ☐ 1. My symptoms get worse<br>☐ 2. No change to my symptoms<br>☐ 3. My symptoms are better |
| Do you tolerate alcohol? | ☐ 1. I can drink and get drunk<br>☐ 2. Drinking alcohol helps me feel better<br>☐ 3. I may have a drink socially<br>☐ 4. I rarely drink alcohol<br>☐ 5. I avoid alcohol because it makes my symptoms worse |

## The Arabic Version of the Questionnaire

1– الموافقة المستنيرة

2– البيانات الديموغرافية

● الجنس ( ذكر / انثي )

● مكان الاقامة

● المستوي التعليمي

● الجنسية ( سعودي / غير سعودي )

الوظيفة

● العمر بالسنوات

● وزن الجسم (كجم) / الطول بالمتر

● الحالة الزوجية ( اعزب / ارمل / متزوج / مطلق )

● الأمراض المرافقة ( (لا شيء، اضطرابات نفسية ونفسولوجية، اضطرابات عصبية، أمراض مناعية ذاتية، مرض السكري، أمراض القلب والشرايين، غيرها)

3– تاريخ الإصابة بفيروس كورونا

● قبل التطعيم، بعد الجرعة الأولى، بعد الجرعة الثانية، بعد الجرعة الثالثة

● خطة الإدارة: (بدون أعراض، في المنزل، تطلب الدخول إلى المستشفى، تطلب الدخول إلى وحد العناية المركزة، تطلب التهوية الميكانيكية

● خطة الإدارة: (بدون أعراض، في المنزل، تطلب الدخول إلى المستشفى، تطلب الدخول إلى وحدة العناية المركزة، تطلب التهوية الميكانيكية

● تم حل الاعراض ( نعم / لا )

4– تاريخ التطعيم ضد فيروس كورونا

● (بيانات الأحداث لكل جرعة (الأولى، الثانية، الثالثة

● سبوتنيك لايت، سيبوتيك-7، سينوفارم، سينوفاك، موديرنا، فايزر،أسترازينيكا، جونسون اند جونسون، مختلطة

● خطة الإدارة: (بدون أعراض، في المنزل، تطلب الدخول إلى المستشفى، تطلب الدخول إلى وحدة العناية المركزة، تطلب التهوية الميكانيكية

● الاثار الجانبية في موضع الحقن ( الألم والثقل والاحمرار والتورم)

● الاثار العامة

● اثار جانبية خطيرة

5– التغذية والمكملات

| | أطلاقا | أقل من ثلاث مرات في الشهر | مرة- مرتين أسبوعيا | أسبوعيا 5-3 | أكثر من خمس مرات أسبوعيا |
|---|---|---|---|---|---|
| الأوميجا ثري او الأسماك التونة المكسرات | | | | | |
| فيتامين دال او التعرض العظام الطويلة (اكثر من ساعه) لأشعة الشمس المباشرة | | | | | |
| اللحوم المصنعة كالهامبرجر و الهوت دوج | | | | | |
| المقليات | | | | | |
| السكريات أكثر من ثلاث ملاعق شاي صغيرة | | | | | |
| تشرب أكثر من لترين ماء يوميا | | | | | |
| التوت او العنب | | | | | |

6– التدريب واللياقة البدنية

| الوصف | المستوي |
|---|---|
| لا امارس أي نشاط بدني عدا الأنشطة اليومية العادية<br>حياة خامله أقل من 5000 خطوة يوميه | حياة خامله |
| اقل من ساعتين ونص في الاسبوع او (من 5000 الي اقل من 7500 خطوه يوميا | منخفض |
| من ساعتين ونص الي اقل من خمس ساعات اسبوعيا او من 7500 الي عشره الالاف خطوه يوميا | متوسط |
| اكثر من خمس ساعات اسبوعيا او اكثر من 10 الف خطوه يوميا | عالي |

7– معدل النوم

● متوسط ساعات النوم

● معدل استمرارية النوم ( متقطع؛ مستمر )

● جودة النوم ( سيئة جدا - سيئة- مقبولة - جيدة - جيدة جدا )

8– معدل وقت تشغيل الشاشة بالساعات

9– تناول الفيتامينات

● الحديد

● الزينك

● فيتامين أ

● فيتامين ب

● فيتامين د

● فيتامين س

● فيتامينات متعددة

● السلينيوم

● الكولاجين

● اخري ( اذكر )

10– استبيان مونتريال التعرفي للنشاط الزهني

النسخة العربية المعدلة من تقييم مونتريال المعرفي    الإسم:    تاريخ الميلاد:    التاريخ:    التعليم:    الجنس (النوع):

**وظائف تنفيذية / وظائف بصرية مكانية** | الدرجات

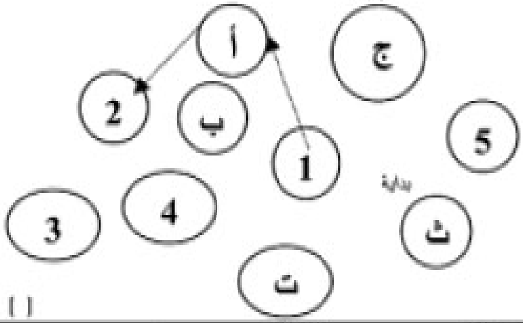


[ ]

ارسم هذا المكعب

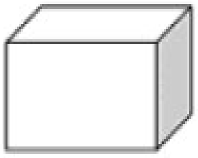

[ ]

ارسم ساعة تشير إلى الساعة 11 و 10دقائق (3 درجات)

[ ] الإطار    [ ] الأرقام    [ ] العقارب

5___

**التسمية**

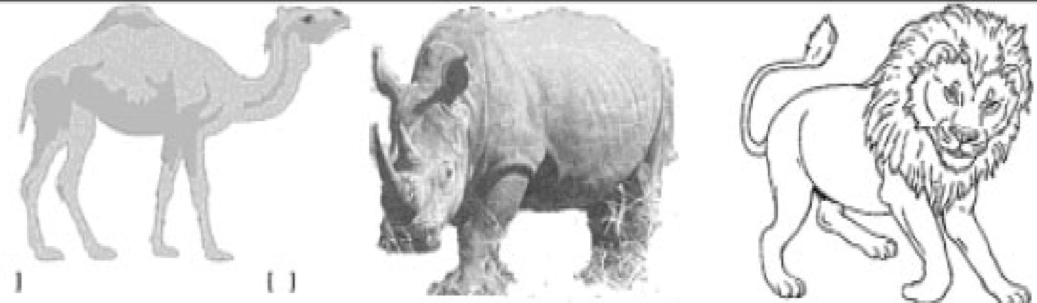

[ ]    [ ]    [ ]

3___

**الذاكرة**

اتلو الكلمات التالية، ثم اطلب من الشخص تلاوتها، قم بإجراء محاولتين. قم باسترجاعهم مرة أخرى بعد خمس دقائق.

| | وجه | ناعم | كنيسة | ممتاز | أحمر |
|---|---|---|---|---|---|
| المحاولة (1) | | | | | |
| المحاولة (2) | | | | | |

لا درجات

**الإنتباه**

إقرأ مجموعة الأرقام (بمعدل رقم في الثانية)

على الشخص أن يكرر تلاوة الأرقام بالترتيب [ ] 21854

على الشخص أن يكرر تلاوة الأرقام عكس الترتيب [ ] 742

2___

إقرأ مجموعة الحروف

على الشخص أن يصفق عند كل حرف "أ"، تعطى درجة "صفر" إذا أخطأ مرتين أو أكثر.

ف ب أ ث م ن أ أ ج ك ل ب أ ف أ ك د ي أ أ أ ج أ م و ف أ أ ب [ ]

1___

اطلب من الشخص أن يقوم بطرح رقم 7 من 100ويكرر عملية الطرح حتى 5 طرحات

[ ] 93    [ ] 86    [ ] 79    [ ] 72    [ ] 65

5-4 طرح صواب : 3 درجة، 3-2 طرح صواب: 2 درجة، 1 طرح صواب: درجة واحدة، لا يوجد صواب: صفر درجة.

3___

**اللغة**

اطلب من الشخص أن يردد الجمل التالية    القرش الأبيض ينفع في اليوم الأسود [ ]    إن غاب القط إلعب يا فأر [ ]

2___

السلاسة في اللغة    اذكر في دقيقة واحدة أكبر عدد من الكلمات تبدأ بحرف الفاء [ ] ____ (≤ 11 كلمة)

1___

**التجريد**

وجه الشبه بين الأشياء مثلاً: الموز و البرتقال = فاكهة

ما وجه الشبه بين:    القطار .. العجلة [ ]    الساعة .. المسطرة [ ]

2___

**الإستدعاء المؤجل**

| | | وجه | ناعم | كنيسة | ممتاز | أحمر | |
|---|---|---|---|---|---|---|---|
| | على الشخص أن يستدعي الكلمات المذكورة من قبل بدون تلميح | [ ] | [ ] | [ ] | [ ] | [ ] | الدرجات للإستدعاء بدون تلميح فقط |
| إختياري | باستخدام التلميح | | | | | | |
| | تلميح باختيارات متعددة | | | | | | |

5___

**التوجه**    [ ] التاريخ    [ ] الشهر    [ ] السنة    [ ] اليوم    [ ] المكان    [ ] المدينة

6___

الدرجة النهائية (الطبيعي) ≥30/26    (أضف درجة إذا كانت عدد سنوات التعليم أقل من12 سنة)    30___

11– استبيان أعراض الإجهاد المزمن ( **English version only available** )

CFS Questionnaire Instructions

Score each symptom in 2 steps. First, has the symptom caused you problems on more than half of the days in the past 6 months? Second, what was the overall severity of the symptom for the past 6 months?

| **Symptom** | **Is this a problem more than half the time?** | | **Severity in the past 6 months** | | | | | **Score** |
|---|---|---|---|---|---|---|---|---|
| | **Yes** | **No** | **None** | **Trivial** | **Mild** | **Moderate** | **Severe** | |
| Fatigue | Yes | No | 0 | 1 | 2 | 3 | 4 | |
| Short term problems with memory or concentrating | Yes | No | 0 | 1 | 2 | 3 | 4 | Sum8 |
| Sore throat | Yes | No | 0 | 1 | 2 | 3 | 4 | |
| Sore lymph nodes (neck, armpits, groin) | Yes | No | 0 | 1 | 2 | 3 | 4 | |
| Muscle pain | Yes | No | 0 | 1 | 2 | 3 | 4 | |
| Joint pain | Yes | No | 0 | 1 | 2 | 3 | 4 | |
| Headaches | Yes | No | 0 | 1 | 2 | 3 | 4 | |
| Difficulty sleeping or unrefreshing sleep | Yes | No | 0 | 1 | 2 | 3 | 4 | |
| Extreme fatigue after exercise or mild exertion | Yes | No | 0 | 1 | 2 | 3 | 4 | |

| | | **Fatigue** | |
|---|---|---|---|
| | | **None, Trivial, Mild** | **Moderate, Severe** |
| Sum of ancillary criteria (Sum8) | 0–13 | ☐ Normal | ☐ Chronic idiopathic fatigue (CIF) |
| | 14–32 | ☐ CFS-like with insufficient fatigue syndrome (CFSLWIFS) | ☐ Chronic fatigue syndrome (CFS) |

| | |
|---|---|
| What would you do if you were better tomorrow? | ☐ 1. I have a list of things to do<br>☐ 2. I would stop being sad<br>☐ 3. Not sure |
| What happens if you walk a long distance? | ☐ 1. My symptoms get worse<br>☐ 2. No change to my symptoms<br>☐ 3. My symptoms are better |
| Do you tolerate alcohol? | ☐ 1. I can drink and get drunk<br>☐ 2. Drinking alcohol helps me feel better<br>☐ 3. I may have a drink socially<br>☐ 4. I rarely drink alcohol<br>☐ 5. I avoid alcohol because it makes my symptoms worse |
